# Quantifying selection bias due to unobserved patients in pharmacoepidemiologic studies of severe COVID-19 cohorts

**DOI:** 10.1186/s12874-025-02732-w

**Published:** 2026-01-16

**Authors:** Marleen Bokern, Christopher T. Rentsch, Jennifer Quint, Anna Schultze, Ian J. Douglas

**Affiliations:** 1 London School of Hygiene and Tropical Medicine, Keppel Street, London, WC1E 7HT UK; 2 Faculty of Medicine, National Heart & Lung Institute, Imperial College London, London, UK

**Keywords:** COVID-19, Pharmacoepidemiology, Respiratory epidemiology, Selection bias, Quantitative bias analysis

## Abstract

**Background:**

The COVID-19 pandemic caused hospital pressures resulting in some patients with severe COVID-19 not being admitted. Studies aiming to measure treatment effects in patients with severe COVID-19 might produce biased estimates if restricted to hospitalised cohorts as a subset of the target population remained unobserved.

**Aim:**

To quantify the effects of potential selection bias due to deaths outside of hospital in a case study of inhaled corticosteroids (ICS) and COVID-19 death among people with chronic obstructive pulmonary disease (COPD) hospitalised with COVID-19.

**Methods:**

Using Clinical Practice Research Datalink Aurum linked to hospitalisation and death registries, we defined a cohort with COPD on 01 Mar 2020, followed up until 31st August 2020. We assessed the odds of COVID-19 death (International Classification of Diseases, 10th Revision U07) among hospitalised COVID-19 patients, comparing current users of ICS/long-acting β-agonist (LABA) and LABA/long-acting muscarinic antagonist (LAMA)). Our target population was those with COPD and severe COVID-19. We evaluated potential selection bias due to non-admission of severe COVID-19 cases using quantitative bias analysis (QBA) in four plausible scenarios, varying assumed death rates among non-hospitalised patients. Selection probabilities for deaths due to COVID-19 were known. The assumptions were: (1) equal odds of death between non-hospitalised and hospitalised groups; (2) doubled odds of death in non-hospitalised ICS/LABA group compared to hospitalised; (3) halved odds of death in non-hospitalised ICS/LABA group; and (4) doubled odds of death in both treatment groups among non-hospitalised patients. We calculated bootstrapped 95% confidence intervals (CIs).

**Results:**

During the study period, 107 ICS/LABA users and 133 LABA/LAMA users were hospitalised with COVID-19. COVID-19 deaths occurred in 42 (39.3%) ICS/LABA users versus 50 (37.6%) LABA/LAMA users. The OR after inverse probability of treatment weighting was 1.01 (95% CI 0.59–1.72). In scenario 1, the OR was unchanged (OR 1.07, 95% CI 0.70–1.67). In scenario 2, the corrected OR was 1.28 (95% CI 0.83–2.00). In scenario 3, the corrected OR was 0.81 (95% CI 0.52–1.23). In scenario 4, the corrected OR was 1.08 (95% CI 0.69–1.71).

**Conclusion:**

QBA facilitated an assessment of the sensitivity of study results to potential selection bias due to non-admission of a subset of patients with severe COVID-19. The results of the four scenarios presented are in line with the null hypothesis, but CIs were wide. Death rates in the non-hospitalised would have needed to be substantially different in the treatment groups to change the study conclusions.

**Supplementary Information:**

The online version contains supplementary material available at 10.1186/s12874-025-02732-w.

## Introduction

The COVID-19 pandemic led to an unprecedented surge in non-interventional studies [1]. Many were designed to assess the impact of established and routinely prescribed medications for other indications to identify possible treatments for patients with severe COVID-19, or to determine whether some ongoing medications might increase the risk of poor outcomes [2]. These studies were often limited to hospitalised patients [3, 4]. It is important to distinguish studies conducted in patients at different stages of COVID-19, from susceptible to infected to severe COVID-19, as treatment effects in patients with severe COVID-19 may differ from effects in the susceptible or infected populations [5]. 

However, treatment effects estimated within hospitalised cohorts can be affected by selection bias if admission is related to the exposure and outcome [6]. Selection bias in this context can arise in two ways in such cohorts. First, conditioning on hospitalisation may induce M-bias by inducing spurious associations between treatment and outcome through shared unmeasured causes of hospitalisation and exposure and outcome, such as disease severity or patient frailty (Supplementary Fig. 2) [6]. This primarily threatens internal validity by distorting treatment effect estimates within the study population. Second, non-admission of patients needing hospitalisation may compromise both generalisability to the target population (all patients with disease enough to warrant hospitalisation) and internal validity if selection creates an imbalance in risk of the outcome between treatment groups within the hospitalised population. Quantitative bias analysis (QBA) methods can provide a way to quantify the impact of biases [7]. However, in practice, these methods are more rarely applied to account for selection bias than for confounding or misclassification [8, 9]. 

This is despite many observational studies starting with a population of patients hospitalised for COVID-19, indicating severe disease [3, 4, 10–12]. In this paper, we evaluated the role of selection bias in a case study investigating whether the routine, ongoing use of inhaled corticosteroids (ICS) amongst hospitalised people with COPD had any effect on severe COVID-19 mortality, aiming to make inference about people with severe COVID-19. During the pandemic in the UK, there were hospital pressures and triaging processes that meant that some people with severe COVID-19 were not admitted to hospital [10, 11]. COVID-19 deaths occurred both in and outside hospitals, indicating that not everyone with severe COVID-19 was hospitalised [12]. When restricting to a hospitalised population, investigators would be missing data on patients with severe COVID-19 who were never hospitalised. To [3, 4] quantify the effects of potential selection bias due to this non-admission of severe COVID-19 cases we used quantitative bias analysis.

## Methods

The original study protocol was registered on ENCEPP EU PAS (register number 47885).

### Data

The data used in this case study came from a previously conducted cohort study, investigating the association between ICS/LABA vs. LABA/LAMA use on the risk of COVID-19 hospitalisation and death [13]. For the purposes of this study, we restricted the study population to hospitalised patients with the aim of investigating the effect of ongoing ICS use at the time of hospital admission on the risk of COVID-19 death. This was used as a case study because it was a common research question and design during the pandemic [3, 4]. The data sources, study population, and exposure, outcome and covariate definitions have previously been described [13], and are summarised below. A table summarising the study design choices is available in Supplementary Table 1.

### Data source

This study used routinely collected primary care data from the UK recorded in Clinical Practice Research Datalink (CPRD) Aurum. CPRD Aurum includes data on 41 million patients (May 2022 build), with over 13 million patients currently registered (20% of the UK population) [14] from >1,300 general practices (GPs) which use EMIS GP patient management software [10]. CPRD Aurum is broadly representative of the English population [15].

CPRD Aurum was linked to Hospital Episode Statistics (HES) Admitted Patient Care (APC) and Office for National Statistics (ONS) Death Registry by NHS Digital using NHS number, sex, date of birth and patient residence postcode [15–17]. HES APC holds information on all in-patient contacts at NHS hospitals in England [16, 18]. The ONS death registry contains information on deaths in England and Wales, including a cause of death documented using International Classification of Disease 10th revision (ICD-10) codes [17, 18]. Data was also linked to the Index of Multiple Deprivation (IMD), a postcode-level indicator of socioeconomic status.

### Study population

We defined a cohort of people diagnosed with COPD before 01 st March 2020 based on a validated algorithm to identify COPD in CPRD using COPD diagnosis codes (PPV 86.5%, 95% CI 77.5% − 92.3%) [19]. Patients were alive and registered in CPRD Aurum on 01 st March 2020, with at least 12 months’ continuous registration prior to this date. In accordance with National Institute for Health and Care Excellence (NICE) guidelines for COPD diagnosis [20], patients needed to be aged ≥ 35 and have a record of current or former smoking at any point before 01 st March 2020. We excluded people with asthma diagnoses recorded within three years, leukotriene receptor antagonist use within 4 months before 01 st March 2020, as this indicates asthma, or other chronic respiratory disease at any point before 01 st March 2020. Follow up for the outcome COVID-19 death (recorded using ICD-10 codes U07.1 and U07.2) began on the date of admission for first COVID-19 hospitalisation. Patients were followed up until death (recorded in ONS or CPRD), deregistration, or 31 st August 2020 (end of first pandemic wave), whichever came first. If death was registered in ONS, we used that date as the date of death. If death was missing in ONS but registered in CPRD, we used the date recorded in CPRD as the date of death. A study diagram [21] is in Supplementary Fig. 1.

### Exposure

Continuous treatment episodes were estimated based on the recorded prescription issue date and information on the intended duration, prescribed amount and dosage (Supplementary Method 1) [13].

We used the derived treatment episodes to categorise people as exposed to ICS/LABA or LABA/LAMA on 01 March 2020 as combined or separate inhalers. People using ICS/LABA/LAMA (i.e., triple therapy) were excluded as we expected patients using triple therapy to be sicker than those on dual therapy. ICS/LABA was the exposure of interest and LABA/LAMA was the active comparator.

### Outcome

The outcome was death with COVID-19 (U07.1 and U07.2) as a cause of death anywhere on the death certificate in the ONS Death Registry, to capture all deaths where COVID-19 was a contributing cause. Given that our study population was restricted to patients hospitalised for COVID-19, it is likely that most such deaths were directly due to COVID-19. Follow-up for the outcome began on the date of COVID-19 hospitalisation.

### Analysis

#### Baseline characteristics and logistic regression models

Cohort characteristics were summarised using descriptive statistics by exposure group. The following covariates were selected for adjustment as potential confounders of the association between treatment and COVID-19 mortality, based on input from a practising clinician: age, sex, body mass index (BMI, most recent within 10 years, categorised as underweight (< 18.5), normal (18.5–24.9), overweight (25–29.9.9), or obese (≥ 30)), smoking (current vs. former), ethnicity, cancer (ever), diabetes (ever), chronic kidney disease (ever), cardiovascular disease (ever), hypertension (ever), asthma (>3 years prior to baseline), immunosuppression, influenza vaccination (past year), pneumococcal vaccination (past 5 years), IMD quintile, and COPD exacerbations in the past 12 mmonths, based on a validated algorithm [22]. 

There were missing data for BMI and ethnicity. Missing BMI was assumed to be normal in line with previous work [23]. Missing ethnicity was treated as a separate category [24].

We estimated propensity scores (PSs) and used inverse probability of treatment weighting (IPTW) to estimate the average treatment effect (ATE) adjusting for potential confounders among those hospitalised for COVID-19. PSs were estimated using logistic regression including the covariates listed above. Stabilised weights were calculated as $\:\frac{P(T=1)}{ps}$ (ICS/LABA) and $\:\frac{P(T=0)}{1-ps}$ (LABA/LAMA), where the PS is the probability of receiving ICS/LABA. Overlap of the PSs across treatment groups was assessed graphically and by summarising PSs by treatment group. PSs were trimmed to the region of common support [25]. 

Logistic regression models were used to estimate odds ratios (ORs) and 95% confidence intervals (CIs), both unweighted and IPT-weighted. This was done to make estimates comparable across analyses, as the correction for selection bias is conducted based on 2 × 2 tables, generating relative risks or ORs as relative effect estimates. 95% CIs were generated using percentile-based bootstrapping using 10,000 iterations. This corresponds to a causal estimand of the average treatment effect (ATE) on the risk of COVID-19 death among patients with severe COVID-19 who were already chronic users of ICS/LABA or LABA/LAMA, conditional on hospitalisation.

#### Selection bias

**Fig. 1:**
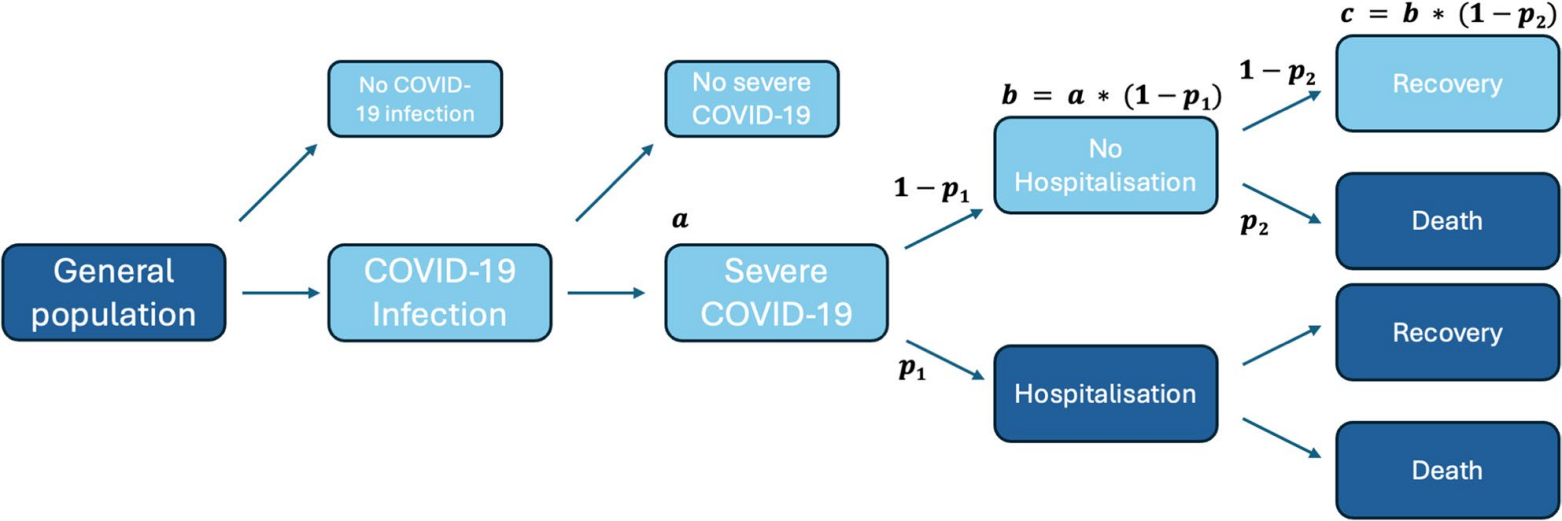
Typical selection pathway in an electronic health record study investigating COVID-19. Dark blue boxes can be observed from data, while light blue boxes remain unobserved. a = number of patients with severe COVID-19, p_1_ = probability of hospitalisation among those with severe COVID-19, b = number of patients with severe COVID-19 who were never hospitalised, p_2_ = probability of death among those with severe COVID-19 who were not hospitalised, c = number of patients with severe COVID-19 who recovered without hospitalisation

Selection into the study followed the flowchart in Fig. 1, which illustrates the situation in a typical electronic health record (EHR) study using population-level data from primary care, hospitalisations and death registries. The target population was people with COVID-19 severe enough to warrant hospitalisation. In the available data, patients in the dark blue boxes could be observed while the light blue boxes were unknown. We observed the number of people in the general population exposed to ICS/LABA or LABA/LAMA, those who became hospitalised, and those who died with or without hospitalisation. If conditioning on hospitalisation leads to systematic differences in terms of outcome risk in the type of patients selected from the ICS/LABA group compared to those selected from the LABA/LAMA group, selection bias may arise due to differences in outcome risk. Differences could arise for various reasons, for example if the occurrence of severe COVID-19 and hospitalisation depends on unobserved factors such as frailty and care home residence [26] that are unequally distributed between treatment groups. A directed acyclic graph (DAG) depicting the assumed structure of selection bias is in Supplementary Fig. 2.

We assumed that all people who were hospitalised or died with COVID-19 had severe COVID-19 and that those with severe COVID-19 who were not hospitalised had COVID-19 severe enough to warrant hospitalisation. Given this, we used the observed odds of death among the hospitalised to set a range of plausible parameters for the odds of death among the non-hospitalised in each treatment group.

#### Correcting for selection bias

Classical correction for selection bias uses the selection probabilities for each combination of exposure and outcome among the target population, which are used to calculate a selection bias odds ratio (sOR) which is applied to the observed effect estimate [7, 27].

1 $$\:\begin{array}{c}sOR=\:\frac{S_{D=1,\:E=1}\ast S_{D=0,E=0}}{S_{D=1,E=0}\ast S_{D=0,E=1}}\:\end{array}$$

2 $$\:\begin{array}{c}{OR}_{adjusted}=\frac{{OR}_{observed}}{sOR}\:\end{array}$$

S is the selection probability, D is the outcome, and E the exposure. In this case, the target population was unobserved, so not all selection probabilities could be directly estimated from the data. In these situations, plausible estimates for selection probabilities in each treatment group could be derived from published literature, clinical experience or representing best- or worst-case scenarios to evaluate the sensitivity of the findings to selection pressures.

In our scenario, plausible ranges for the selection probabilities could be calculated after estimating the hospitalisation probability among those with severe COVID-19 (p_1_, Fig. 1), the number of people with severe COVID-19 (a), the number of people with severe COVID-19 not hospitalised (b), or the probability or odds of death outside of hospital (p_2_). As we had data on deaths without preceding hospitalisation, we could calculate the selection probabilities for COVID-19 deaths and needed to estimate only the selection probabilities for the recovered.

As we observed the odds of death among the hospitalised, we estimated the odds of death among the non-hospitalised by treatment group. This allowed us to calculate the number of recovered non-hospitalised patients as

3 $$\:\begin{array}{c}n_{D=0,\:\:H=0}=\frac{n_{D=1,\:H=0}}{{odds}_{D=1\vert H=0}}\:\end{array}$$.

N represents numbers of patients, D is the outcome, and H is hospitalisation. We then calculate the number of people with severe COVID-19 by treatment group by adding the unobserved number of recovered non-hospitalised patients $\:{n}_{D=0,\:\:H=0}$ to the observed total deaths and the observed number of hospitalised recovered. This allows us to calculate the selection probabilities and to correct the observed OR for selection bias (Eq. 1).

We calculated ORs varying the odds of death among the non-hospitalised between 0.05 and 2.0 in each treatment group in 0.05 increments. Odds of death among the hospitalised are known from the data. We depict the results of this analysis with a heat map.

Additionally, we highlight 4 scenarios assuming odds of death among the non-hospitalised. Percentile-based 95% CIs were estimated using bootstrapping with 10,000 iterations.

##### Scenario 1: non-hospitalised same as hospitalised

The odds of death, by treatment group, are the same among those who were not hospitalised as among those who were hospitalised. This means that selection probabilities were assumed to be equal for unobserved exposed and unexposed non-cases. This represents a situation where hospitalisation had no impact on survival and theoretically, we would expect this to result in zero bias.

##### Scenario 2: ICS/LABA non-hospitalised sicker than LABA/LAMA non-hospitalised

The odds of death in the LABA/LAMA group are the same among those who were not hospitalised as among those who were hospitalised but are doubled in the ICS/LABA group among those not hospitalised compared with those hospitalised. This models a scenario where non-hospitalised ICS/LABA patients are sicker than non-hospitalised LABA/LAMA patients. Alternatively, it can be thought of as setting higher selection probabilities for exposed COVID-19 deaths compared to unexposed COVID-19 deaths.

##### Scenario 3: ICS/LABA non-hospitalised healthier than LABA/LAMA non-hospitalised

The odds of death in the LABA/LAMA group are the same among those who were not hospitalised as among those who were hospitalised, but are halved in the ICS/LABA group among those not hospitalised compared with those hospitalised. This models a scenario where non-hospitalised ICS/LABA patients are healthier than non-hospitalised LABA/LAMA patients.

##### Scenario 4: non-hospitalised sicker than hospitalized

In both treatment groups, the odds of death are doubled among those who were not hospitalised compared to those who were hospitalised.

As a diagnostic check, we calculated the other unknown quantities in Fig. 1 for scenarios 1–4 by treatment group. The total number of non-hospitalised patients with severe COVID-19 was calculated by summing the previously calculated number of patients with severe COVID-19 who were not hospitalised and recovered and the total number of COVID-19 deaths outside of hospital.

4 $$\:\begin{array}{c}n_{H=0}=\:n_{H=0,\:D=0}+\:n_{H=0,\:D=1}\end{array}$$

Thereupon, the total number of people with severe COVID-19 was calculated as the calculated number of non-hospitalised patients with severe COVID-19 plus the hospitalised.

5 $$\:\begin{array}{c}n=\:n_{H=0}+\:n_{H=1}\end{array}$$

Finally, the corresponding probability of hospitalisation was calculated as the hospitalised divided by the assumed total number of patients with severe COVID-19.

6 $$\:\begin{array}{c}p_{H=1}=\frac{n_{H=1}}n\:\end{array}$$

Based on Eq. 6, we calculate the upper bound for the selection probabilities are equal to the number of hospitalisations divided by the number of hospitalisations plus the number of deaths outside of hospital.

Data was managed using Stata MP version 17.0 [28] and analysis carried out using R (version 4.4.2) [29]. Code lists and data management and analysis code are on GitHub (https://github.com/bokern/ics_covid_collider).

## Results

### Analysis restricting to hospitalised patients

The hospitalised COPD cohort consisted of 107 ICS/LABA users and 133 LABA/LAMA users (Table 1). The median time to hospitalisation was 38 days (IQR 29–62.2 days) from 01 March 2020. In the ICS/LABA group, 42 (39.3%) experienced the outcome COVID-19 death, compared with 50 (37.6%) in the LABA/LAMA group.

**Table 1:** Baseline cohort characteristics before and after inverse probability of treatment weighting

	Unweighted			IPT-weighted		
	ICS *N* = 107	LABA/LAMA *N* = 133	SMD	ICS *N* = 107	LABA/LAMA *N* = 133	SMD
Age			0.020			0.029
*Mean (SD)*	78.1 (11.2)	77.8 (9.9)		77.62 (11.15)	77.92 (9.71)	
*Median (Q1-Q3)*	78.7 (71.7–85.7)	79.7 (72.7–83.7)		78.67 (70.67–84.67)	79.67 (72.67–83.67)	
Sex			0.092			0.004
*Male*	63 (59%)	90 (68%)		69 (65%)	86 (64%)	
*Female*	44 (41%)	43 (32%)		38 (35%)	47 (36%)	
BMI						
*Underweight (< 18.5)*	3 (2.8%)	4 (3.0%)	0.006	24 (23%)	32 (24%)	0.001
*Normal (18.5–24.9)*	26 (24%)	32 (24%)	0.010	2 (2.2%)	3 (2.3%)	0.010
*Overweight (25–29.9.9)*	40 (37%)	45 (34%)	0.044	39 (36%)	49 (37%)	0.021
*Obese (≥ 30)*	38 (36%)	52 (39%)	0.029	42 (39%)	49 (37%)	0.011
Ethnicity						
*White*	87 (81%)	118 (89%)	0.048	94 (88%)	116 (87%)	0.002
*South Asian*	4 (3.7%)	1 (0.8%)	0.002	1 (1.1%)	1 (1.1%)	< 0.001
*Black*	3 (2.8%)	3 (2.3%)	0.006	2 (2.1%)	3 (2.0%)	0.001
*Mixed*	1 (0.9%)	1 (0.8%)	0.002	1 (0.9%)	1 (0.9%)	< 0.001
*Unknown*	12 (11%)	10 (7.5%)	0.038	9 (8.2%)	11 (8.5%)	0.003
Smoking			0.052			0.009
*Current smoking*	27 (25%)	27 (20%)		25 (23%)	32 (24%)	
*Former smoking*	80 (75%)	106 (80%)		82 (77%)	101 (76%)	
Index of Multiple Deprivation						
*1 (most deprived)*	7 (6.5%)	18 (14%)	0.048	12 (11%)	13 (9.7%)	0.013
*2*	11 (10%)	24 (18%)	0.079	17 (15%)	20 (15%)	0.002
*3*	22 (21%)	24 (18%)	0.027	21 (19%)	26 (20%)	0.004
*4*	36 (34%)	41 (31%)	0.079	33 (31%)	44 (33%)	0.009
* 5 (least deprived)*	31 (29%)	26 (20%)	0.048	25 (23%)	30 (22%)	0.013
Diabetes	36 (34%)	50 (38%)	0.042	36 (33%)	45 (34%)	0.007
Hypertension	74 (69%)	82 (62%)	0.062	69 (65%)	88 (66%)	0.013
Cardiovascular disease	56 (52%)	69 (52%)	0.012	52 (49%)	66 (50%)	0.009
Cancer	29 (27%)	28 (21%)	0.062	27 (25%)	32 (24%)	0.006
Past asthma	24 (22%)	13 (9.8%)	0.102	15 (14%)	19 (14%)	0.002
Kidney impairment	57 (53%)	80 (60%)	0.069	60 (56%)	76 (57%)	0.009
Immunosuppression	2 (1.9%)	1 (0.8%)	0.012	1 (1.2%)	1 (1.1%)	0.001
Influenza vaccine	86 (80%)	116 (87%)	0.071	89 (83%)	110 (83%)	0.004
Pneumococcal vaccine	9 (8.4%)	12 (9.0%)	0.006	8 (7.7%)	11 (8.4%)	0.007
Any exacerbation in past 12 months	46 (43%)	54 (41%)	0.025	42 (40%)	54 (41%)	0.007

*BMI* Body mass index, *IPT* Inverse probability of treatment, *SD* Standard deviation, *SMD* Standardised mean difference

**Fig. 2:**
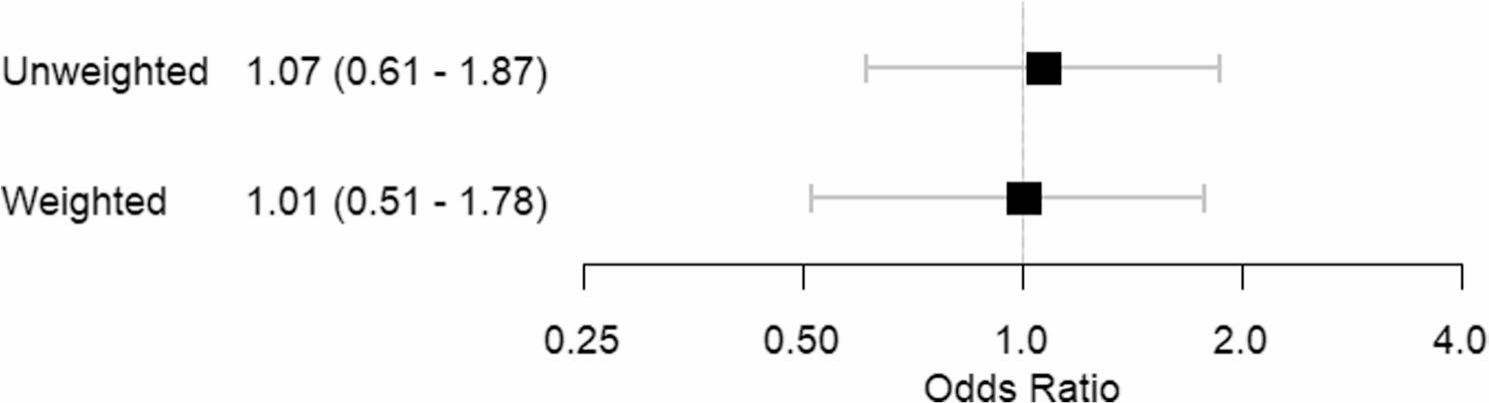
Results of logistic regression models with 95% confidence intervals before and after inverse probability of treatment weighting. Confidence intervals were constructed using percentile-based bootstrapping (*n* = 10,000 iterations). Scenario 1: no differential bias (odds of death equal between non-hospitalised and hospitalised in both treatment groups). Scenario 2: non-hospitalised ICS/LABA users are sicker (odds of death doubled compared to hospitalised ICS/LABA), reflecting higher selection probability for exposed deaths. Scenario 3: non-hospitalised ICS/LABA users are healthier (odds of death halved). Scenario 4: non-hospitalised individuals in both groups are generally sicker (odds of death doubled compared to hospitalised)

Trimming the cohort to the region of common support did not exclude many patients (*n* = 6 or 2.5% of the total study population, 3 from each treatment group) Using logistic regression, the unweighted OR was 1.07 (95% CI 0.63–1.81), which moved towards the null after IPTW (OR 1.01 (95% CI 0.59–1.72)) (Fig. 2).

### Selection bias

**Fig. 3:**
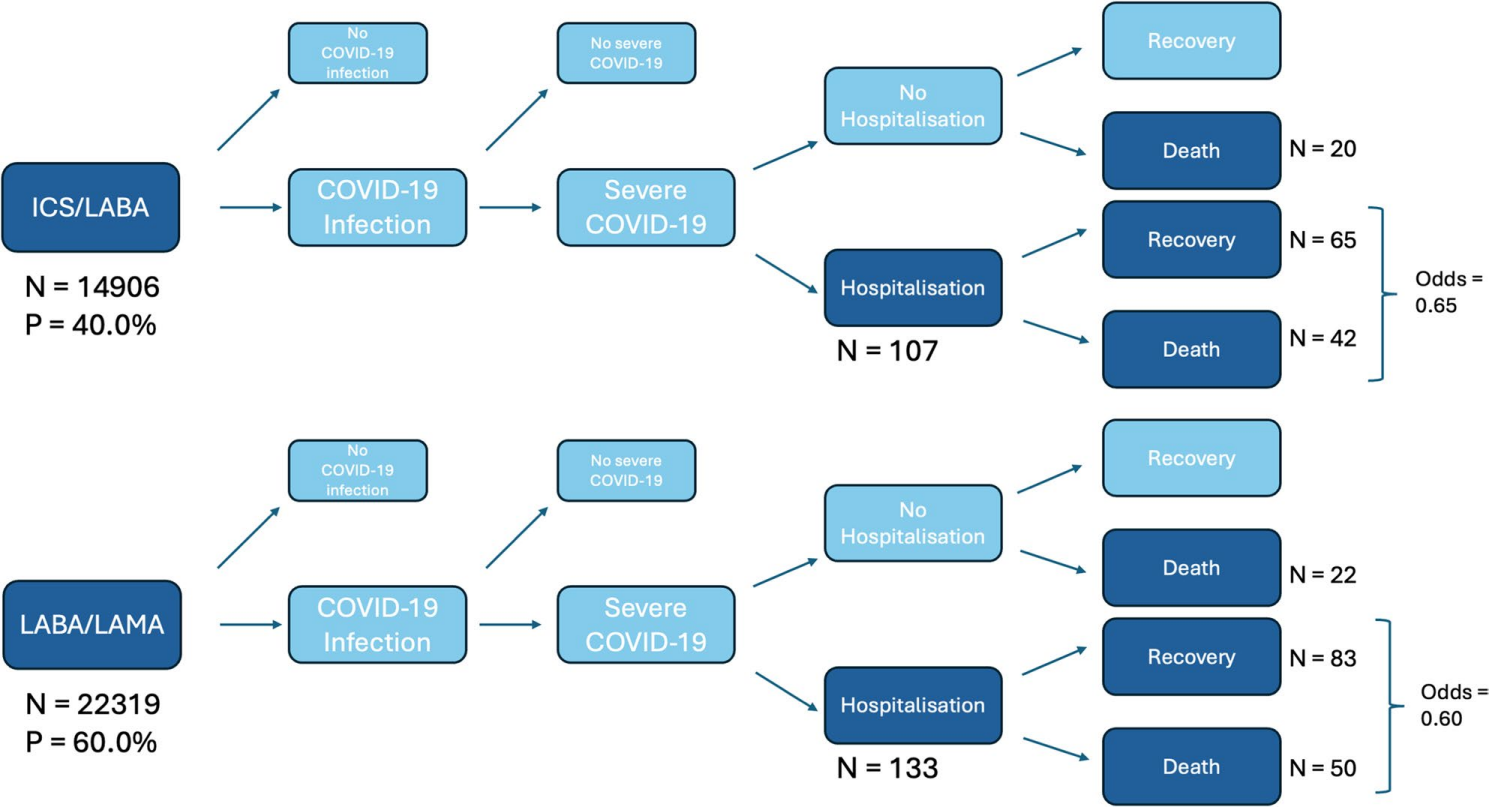
Flowchart of patient selection. “Severe COVID-19” means COVID-19 severe enough to warrant hospitalisation. Dark blue boxes represent populations we can observe from the available data. Light blue boxes cannot be observed

There were 20 deaths without hospitalisation in the ICS/LABA group and 22 in the LABA/LAMA group (Fig. 3). Among those hospitalised, the odds of death were 0.65 in the ICS/LABA group and 0.60 in the LABA/LAMA group.

**Fig. 4:**
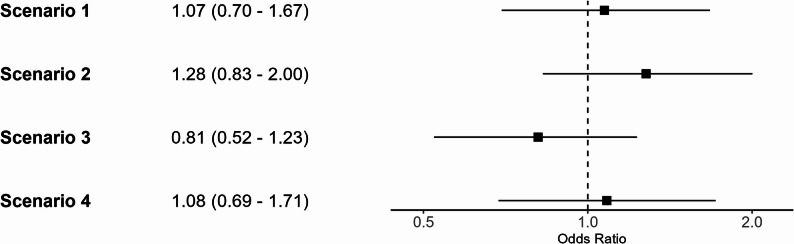
Forest plot of odds ratios and 95% confidence intervals after correction for selection bias. CIs were generated using percentile-based bootstrapping (*n* = 10,000 iterations)

**Fig. 5:**
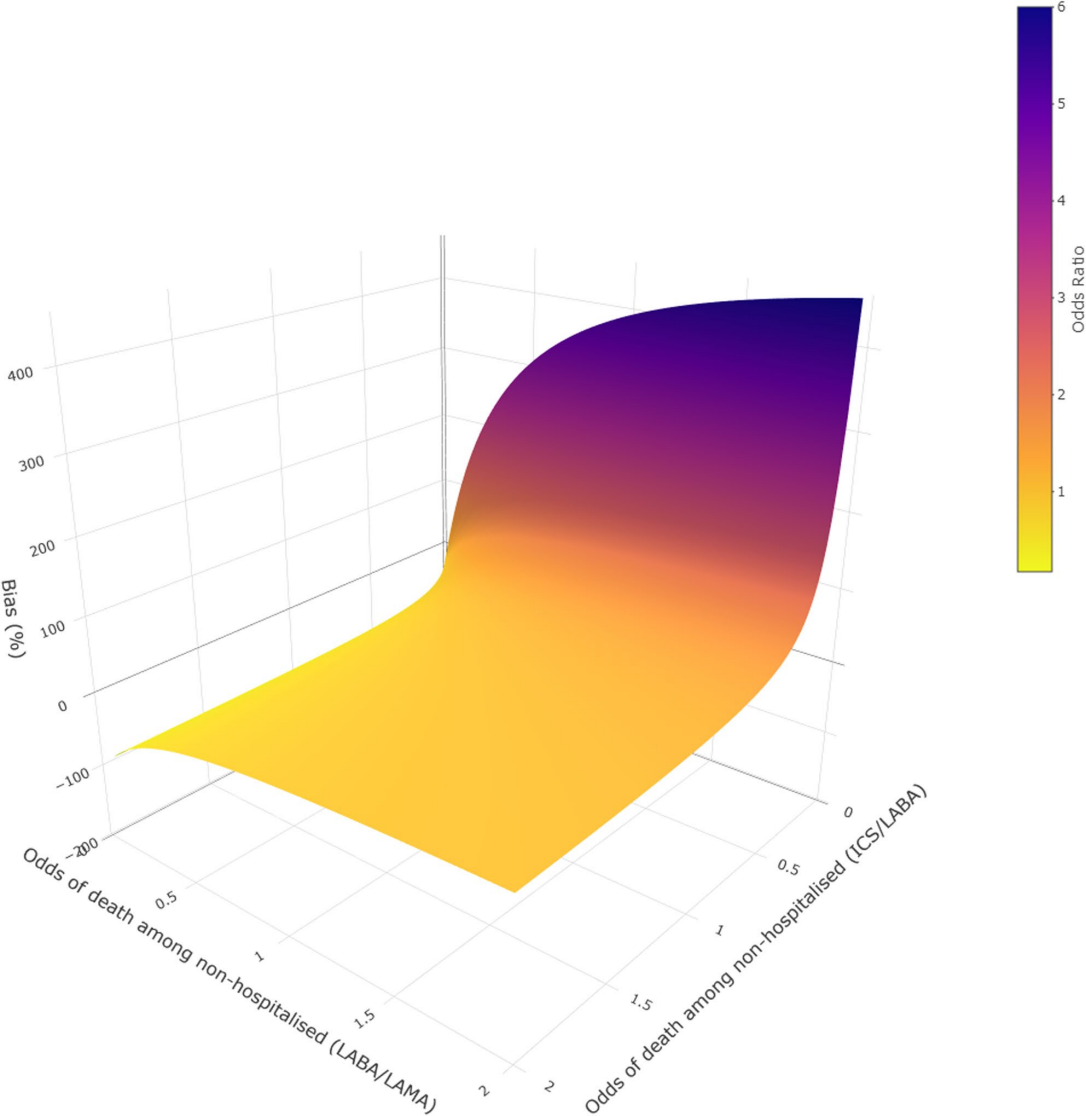
Three-dimensional surface plot depicting odds ratios and corresponding percentage bias corrected for differential odds of death among the (unobserved) non-hospitalised patients with severe COVID-19 in the ICS and LABA/LAMA groups

Table 2 presents the results of scenarios 1–4. ORs vary between 0.81 and 1.27, with all 95% CIs crossing the null (Fig. 4), with corresponding probabilities of hospitalisation among those with severe COVID-19 between 0.57 and 0.77 (Supplementary Table 2). When varying the assumed odds of death incrementally, the ORs for most combinations of odds of death were similar to the observed OR, with only very low odds of death in one of the treatment groups resulting in large changes to the OR (Fig. 5). For the ICS/LABA group, the upper bound for the probability of hospitalisation among those with severe COVID-19 was 0.843, and for the LABA/LAMA group, it was 0.858.

**Table 2:** Results of scenarios 1–4

	ICS		LABA/LAMA		
Scenario	Odds of death among non-hospitalised	Odds of death among severe COVID-19	Odds of death among non-hospitalised	Odds of death among severe COVID-19	Odds ratio
1	0.65	0.65	0.60	0.60	1.07
2	1.29	0.77	0.60	0.60	1.28
3	0.32	0.49	0.60	0.60	0.81
4	1.29	0.77	1.20	0.71	1.08

## Discussion

We have applied a method to account for potential selection bias in a study of COVID-19 mortality estimating treatment effects among individuals with severe COVID-19 using a hospitalised cohort. After correcting for the potential selection bias, the adjusted OR for COVID-19 death comparing ICS/LABA users with LABA/LAMA users was similar to the observed OR for many combinations of selection probabilities. For four pre-defined plausible scenarios, ORs varied between 0.81 when the odds of death among the unobserved in the ICS/LABA group were halved compared with the observed, and 1.28 when the odds of death among the unobserved in the ICS/LABA group were doubled compared with the observed. However, 95% CIs calculated using bootstrapping overlapped with a null effect under all scenarios. For the conclusions to have substantially differed from those of the observed results, the odds of death among unobserved ICS/LABA patients would have needed to be either less than half or more than double compared to the observed.

### Comparison with previous work

Selection bias has not frequently been addressed in pharmacoepidemiologic studies [8, 9]. Some examples come from the field of perinatal pharmacoepidemiology. A study investigating selective serotonin reuptake inhibitor use in pregnancy and cardiac defects assessed potential selection bias due to missing terminations, correcting the observed OR using selection probabilities for each exposure-outcome combination [30]. Further studies investigating lithium use and cardiac malformations and statins and congenital malformations used similar methods [27, 31]. One study investigated selection bias in a study of allergy medications and COVID-19 testing [32]. The authors used a bounding factor [33] to calculate the smallest the true OR could be given assumed risk (or odds) ratios between a selection factor and the exposure and outcome. This method can be used when correcting for collider bias where the selected population is the target population [32, 33]. 

Regarding the clinical question, two previous studies investigating the effects of ICS on COVID-19 outcomes among hospitalised patients, both suggesting no effect of ICS. In a cohort of COPD patients hospitalised with COVID-19 in Denmark, routine use of ICS was not associated with an increased risk of death (HR 1.02 (95% CI, 0.78–1.32)) compared to bronchodilator use. In a hospitalised UK cohort, among those with chronic pulmonary disease, both those with and without routine ICS use had an increased hazard of death with COVID-19 (no ICS use: HR 1.16, 95% CI 1.12–1.22; using ICS: HR 1.10, 95% CI 1.04–1.16) compared to patients without respiratory disease [3]. As this study was also set in the UK during the first wave of the pandemic, selection pressures among people with respiratory diseases would have been similar to this study. However, because the comparator group did not have respiratory disease, the findings are not directly comparable to ours.

### Interpretation

We illustrate that selection bias is introduced if there are differential selection pressures into hospital on the exposed and unexposed groups. This may be less likely in pharmacoepidemiological studies using an active comparator and is confirmed with very similar selection probabilities for cases in the exposed and unexposed cases (0.68 in the ICS/LABA group and 0.69 in the LABA/LAMA group).

While the point estimates vary substantially when varying selection probabilities, differences in selection probabilities for the non-cases as large as assumed in scenarios 2 and 3 may be unrealistic given the treatment groups were relatively similar (Table 1). Furthermore, one may assume that hospitalisation would reduce the risk of death compared to those with severe COVID-19 who were not admitted, making scenarios 2 and 4 potentially more likely than scenarios 1 and 3. For all four calculated scenarios, the CIs are in line with the null hypothesis and the uncorrected result.

For a mechanism to introduce bias, either the selection probabilities for non-cases need to differ for exposed and unexposed individuals or, equivalently, the recovery rates for the non-hospitalised need to differ between treatment groups. Hospital pressures during the pandemic meant that some people with severe COVID-19 may not have been admitted to hospital if they were deemed unlikely to survive, as resources would have been preferentially allocated to patients with higher survival probability [2]. 

We used an active comparator design to minimise differences between our comparison groups. Traditionally, this approach is used to deal with confounding. A further benefit of use of active comparators is to reduce differences in selection probabilities by ensuring comparison groups have similar background illnesses and are therefore treated more similarly in a health and social care system. They can therefore also help mitigate the risk of selection bias [34]. Much of the literature on QBA for selection bias does not take active comparators into account [6, 35]. 

In this study, the probability of being hospitalised with COVID-19 may depend on place of residence (e.g., care home vs. private homes) and frailty. In these datasets, care home residence is not readily available, and frailty can be difficult to ascertain in EHRs [36]. Hospital admissions from care homes dropped at the start of the pandemic [37]. Additionally, people with COVID-19 were discharged to care homes, causing outbreaks within care homes [38]. Taken together, people living in care homes may have been less likely to be admitted to hospital with severe COVID-19 and therefore may be more likely to be missed when restricting to hospitalisation. Such a mechanism could underpin different probabilities of hospital admission between comparison groups, and the extent of these differences would then influence the degree to which selection bias may lead to incorrect results.

Our study design does not explicitly emulate a clinical trial of drug initiation or discontinuation. Such studies would not be feasible to emulate during the first wave of COVID-19 due to the short study period, with few people initiating chronic medications and discontinuations being hard to ascertain accurately. The effect estimates in this study might approximate those from an emulated trial of drug initiation under the assumption of no depletion of susceptibles, which seems reasonable given that COVID-19 did not exist prior to 1 March 2020 [39]. However, our primary aim was to evaluate selection bias rather than establish treatment effects for clinical decision making. We note that an emulated target trial restricted to a hospitalised population would still be subject to selection bias even if the target population were hospitalised patients and we had pre-specified explicit treatment strategies involving either initiation or discontinuation shortly before or during hospitalisation. The study design we used was commonly employed by studies during the COVID-19 pandemic and might be understood as informing mortality risk among chronic users of different treatments without necessarily corresponding to specific, clearly defined treatment strategies around drug initiation or discontinuation. The target population in our study was people with COVID-19 severe enough to warrant hospitalisation, with hospital admission used as a proxy for severe disease. The target population determines which methods to account for selection bias may be appropriate. In this case, reweighting our results to the whole population would reproduce results for the full population of people with COPD, not the population with severe COVID-19 [40]. Simply not restricting analyses to hospitalised people would avoid selection bias, but would also change the target population to the whole population. This would address a different question and illustrates why articulation of the target population is key.

### Strengths and limitations

Strengths of this study include the representativeness of CPRD Aurum, as well as the comprehensive capture of hospitalisations and deaths.

Exposures were defined on 01 March 2020 whereas follow-up for the outcome began at hospitalisation. This assumes that patients remained on the same treatment between 01 March and hospitalisation. Due to medication stockpiling [3, 41] and disruptions to healthcare services [42] during the pandemic, discontinuations are difficult to ascertain in the study period [39]. Due to the short study period, this misalignment likely causes only a small amount of misclassification.

Similarly, we assessed confounders on 01 March 2020 but follow-up did not begin until hospitalisation, thus some misclassification might be possible (e.g., COPD exacerbations) if these variables changed substantially over the 6 month study period.

Our analysis includes prevalent users, which means that the treatment groups differ in prior exposure history. A new-user design would be preferable for causal inference. However, during the short study period (March–August 2020), there were few new initiators of either therapy, making an ACNU design infeasible. Because these medications are used chronically, a prevalent-user design captures the experience of real-world users during the early pandemic.

QBA is not frequently used in non-interventional research, and QBA for selection bias is particularly rare [8, 9]. This may be because quantifying selection probabilities is difficult in many situations. We have illustrated how these methods can be implemented with reference to odds of the outcome, as opposed to sampling probabilities, which may be easier for researchers to estimate. Illustrating the selection process using a flowchart requires researchers to think through selection processes and may further aid in the application of QBA. In the absence of well-informed estimates of selection probabilities, heat maps can show a wide range of assumptions simultaneously.

The correction method presented here does not attempt to account for residual confounding. Doing so would require more complex modelling or simulation methods and therefore not be pragmatic to implement for many researchers. Alternatively, selection probabilities or odds of death can be estimated within strata of confounders [43]. However, conventional analyses weighted by PS showed little change in OR both when restricting to hospitalised patients, and in the whole COPD cohort [13]. While this is not proof that confounding did not affect analyses among people with severe COVID-19, this is reassuring regarding the suitability of the active comparator.

The flowchart (Fig. 1) is specific to this time period in the UK when COVID-19 testing was limited, and we therefore treat COVID-19 infections as missing. In other settings where virus circulation is lower or testing more widely available, test data may provide a reliable estimate of infections, and therefore of those susceptible to severe COVID-19. In an acute pandemic scenario, it may also be more feasible to assess the selection probabilities by making assumptions on probability of infection and severe disease rather than survival outcomes. The choice of parameters to estimate may depend on the nature of the selection process and what data is available at different time points.

Hospital pressures leading to non-admission of severe COVID-19 cases may not have been consistent throughout the follow-up time. Most deaths and hospitalisations occurred between late March and mid-May. Bias parameters may therefore have changed over the study period, with more impactful hospital pressures in the first half of our study period. However, we assume that changes to hospital pressures over the study period would have affected both treatment groups similarly.

Our approach of calculating recovery rates relies on data on deaths outside of hospitals, so selection probabilities for people with the outcome were known. This may be the case if the outcome is ascertained through linked data. If these probabilities were unknown, more assumptions would have to be made (e.g. odds of death among the non-hospitalised, and the number of either deaths or recoveries outside of hospital by treatment group). It is difficult to ascertain the plausibility of different assumptions regarding the odds of death among the non-hospitalised. However, we consider large deviations, such as less than half or more than double of those observed, unlikely given the comparability of the treatment groups at baseline (Table 1).

We focus on selection bias due to missing data. However, even if all severe COVID-19 cases were hospitalised and selected into the study, collider bias may still distort associations between factors that cause hospitalisation and should be dealt with as a separate consideration [6, 26]. Methods to account for selection bias in this special case where the target population is the selected population have overlap with methods to adjust for confounding [44]. We chose an active comparator that minimises confounding, which simultaneously reduces selection bias.

In summary, we demonstrate the use of QBA to correct for assumed differential selection probabilities in a pharmacoepidemiological study. While all QBA is based on assumptions, our study highlights the value of carefully considering the source and structure of selection bias, and attempting to quantify its potential impact. In this example, we demonstrated that plausible differences in selection probabilities were unlikely to have changed the conclusions of the study.

## Supplementary Information

Supplementary Material 1.

Supplementary Material 2.

## Data Availability

No additional data is available. Data management and analysis code, along with all code lists, are available on our GitHub repository (https://github.com/bokern/ics_covid_collider).
