# Supplementary material for: Quantifying selection bias due to unobserved patients in pharmacoepidemiologic studies of severe COVID-19 cohorts
Source: BMC Med Res Methodol. 2026 Jan 16;26:34. doi: 10.1186/s12874-025-02732-w (PMC12896000; doi:10.1186/s12874-025-02732-w)
Supplement: Supplementary file 1 — Supplementary Material 1. [file 12874_2025_2732_MOESM1_ESM.pdf]

Supplementary material: Quantifying selection bias due to unobserved patients in pharmacoepidemiologic studies of severe COVID-19 cohorts.

Marleen Bokern[a*], Christopher T. Rentsch[a], Jennifer Quint[b], Anna Schultze[a], Ian Douglas[a]

*Corresponding author; email: marleen.bokern@lshtm.ac.uk

[a] London School of Hygiene and Tropical Medicine, Keppel Street, London WC1E 7HT, UK

[b] Faculty of Medicine, National Heart & Lung Institute, Imperial College London, London, UK

Supplementary Figure 1 Study diagram 2

Supplementary Figure 2 Directed acyclic graph (DAG) depicting the assumed structure of selection bias 3

Supplementary Figure 3 Unweighted propensity score distribution 3

Supplementary Figure 3 Propensity score distribution after inverse probability of treatment weighting 4

Supplementary Figure 3 Absolute standardised mean differences (SMDs) before and after inverse probability of treatment weighting 5

Supplementary Method 1 Example calculation, scenario 1 6

Supplementary Table 1 2x2 table of hospitalisation and death for ICS group 6

Supplementary Table 2 2x2 table of hospitalisation and death for LABA/LAMA group 7

Supplementary Table 3 Diagnostic checks of scenarios 1-4. 8

Supplementary Figure 1 Study diagram

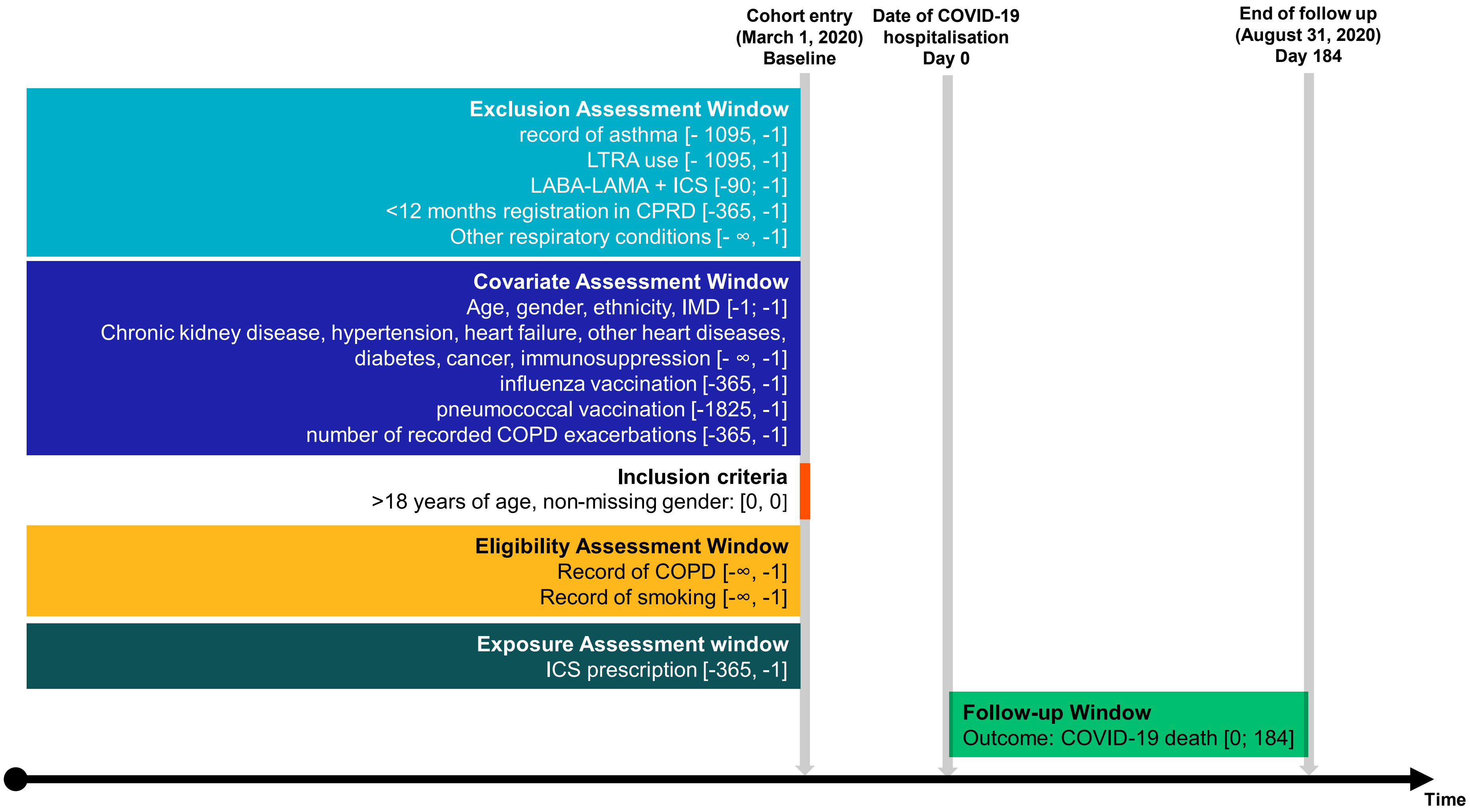

Supplementary Figure 2 Directed acyclic graph (DAG) depicting the assumed structure of selection bias

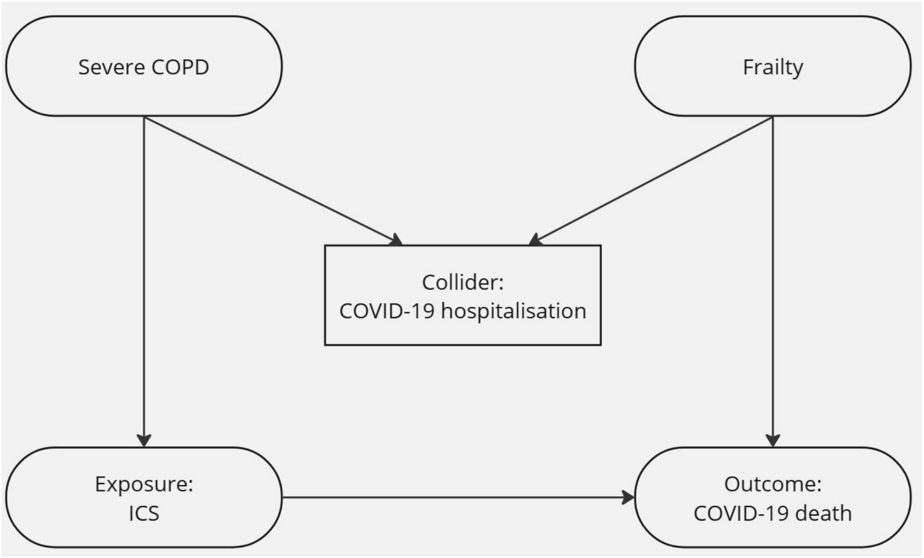

Supplementary Figure 3 Unweighted propensity score distribution

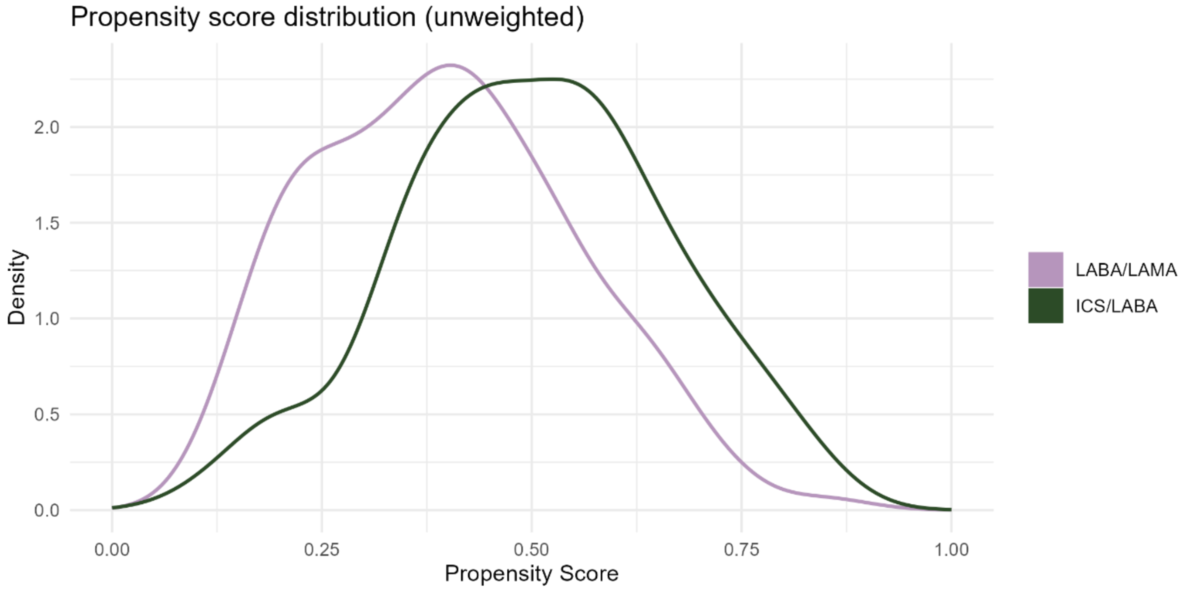

Supplementary Figure 3 Propensity score distribution after inverse probability of treatment weighting

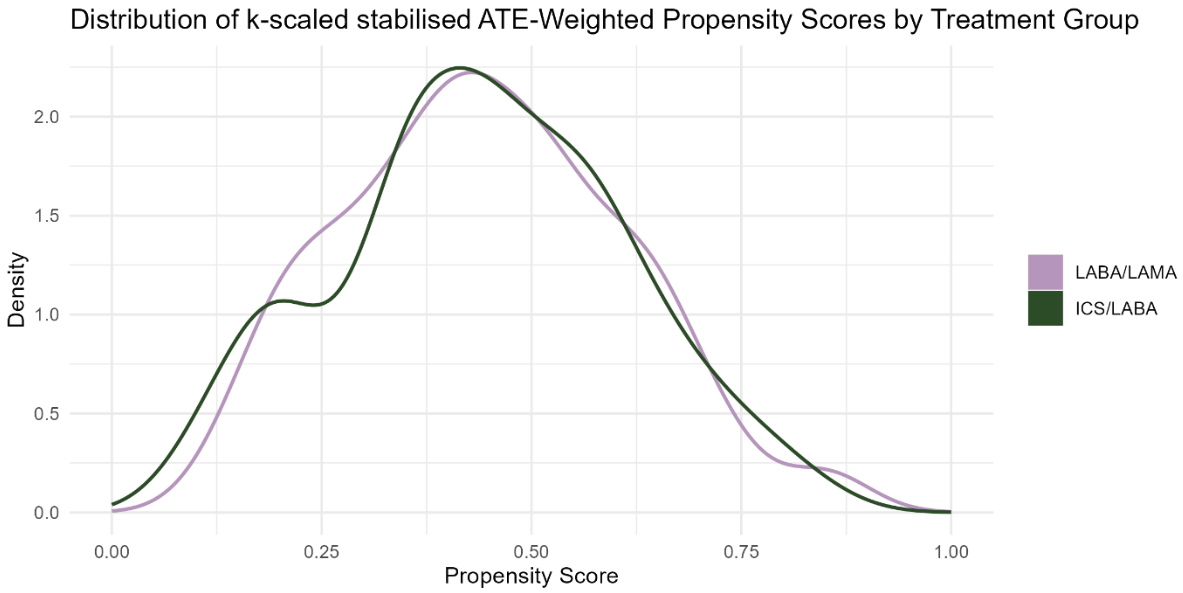

Supplementary Figure 3 Absolute standardised mean differences (SMDs) before and after inverse probability of treatment weighting

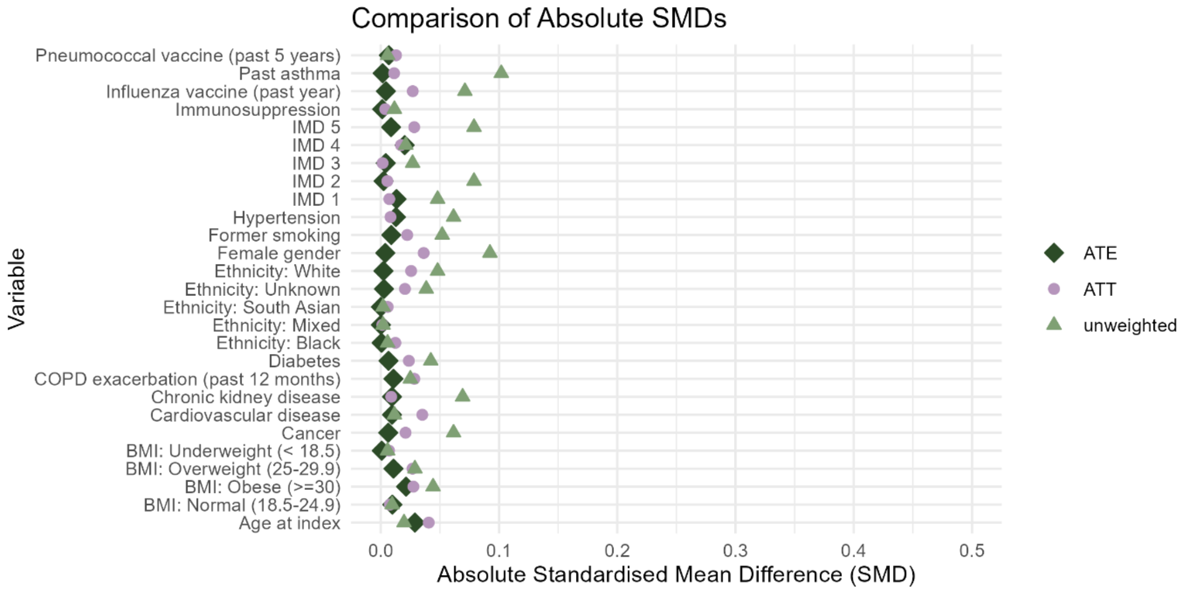

Supplementary Method 1 Example calculation, scenario 1

The odds of death in the hospitalised for each treatment group is as follows:

$$odds_{D=1|E=1,H=1} = \frac{n_{D=1,E=1,H=0}}{n_{D=0,E=1,H=0}} = \frac{42}{65} = 0.65 \quad (1)$$

$$odds_{D=1|E=0,H=1} = \frac{n_{D=1,E=0,H=0}}{n_{D=0,E=0,H=0}} = \frac{50}{83} = 0.60 \quad (2)$$

We assume the odds of death are the same among the non-hospitalised compared to the hospitalised (scenario 1).

As we have data on the number of COVID-19 deaths outside of hospitals by treatment group, we calculate the number of patients with severe COVID-19 who recovered outside of hospital.

$$n_{D=0,E=1,H=0} = \frac{n_{D=1,E=1,H=0}}{odds_{D=1,E=1,H=1}} = \frac{20}{0.65} = 30.95 \approx 31 \quad (3)$$

$$n_{D=0,E=0,H=0} = \frac{n_{D=1,E=0,H=0}}{odds_{D=1,E=0,H=1}} = \frac{22}{0.60} = 36.52 \approx 37 \quad (4)$$

Adding together the observed hospitalisations, the COVID-19 deaths without hospitalisation and the assumed number of recoveries without hospitalisation, we have 158 patients with severe COVID-19 in the ICS group and 192 in the LABA/LAMA group.

Having calculated the number of people with severe COVID-19 who recovered, we can calculate an odds ratio accounting for the people we did not observe in the hospitalisation data.

$$OR = \frac{n_{D=1,E=1} * n_{D=0,E=0}}{n_{D=1,E=0} * n_{D=0,E=1}} = \frac{62 * (83 + 37)}{72 * (65 + 31)} = 1.07 \quad (5)$$

Supplementary Table 1 2x2 table of hospitalisation and death for ICS group

ICS group		Hospitalisation		
		Hospital	No hospital	
Death	Death	$n_{D=1, H=1}$ = 42	$n_{D=1, H=0}$ = 20	$n_{D=1}$ = 62
	Survived	$n_{D=0, H=1}$ = 65	$n_{D=0, H=0}$ = ?	$n_{D=0}$ = ?
		$n_{H=1}$ = 107	$n_{H=0}$ = ?	

Supplementary Table 2 2x2 table of hospitalisation and death for LABA/LAMA group

LABA/LAMA group		Hospitalisation		
		Hospital	No hospital	
Death	Death	$n_{D=1, H=1}$ = 50	$n_{D=1, H=0}$ = 22	$n_{D=1}$ = 72
	Survived	$n_{D=0, H=1}$ = 83	$n_{D=0, H=0}$ = ?	$n_{D=0}$ = ?
		$n_{H=1}$ = 133	$n_{H=0}$ = ?	

Supplementary Table 3 Diagnostic checks of scenarios 1-4. For totals, decimals were rounded up to the nearest integer.

	ICS					LABA/LAMA				
Scenario	Odds of death among non-hospitalised	n (severe COVID-19, not hospitalised, recovered)	n (severe COVID-19, not hospitalised)	n (severe COVID-19)	p (hospitalisation)	Odds of death among non-hospitalised	n (severe COVID-19, not hospitalised, recovered)	n (severe COVID-19, not hospitalised)	n (severe COVID-19)	p (hospitalisation)
1	0.65	31	51	158	0.68	0.60	37	59	192	0.69
2	1.29	16	36	143	0.75	0.60	37	59	192	0.69
3	0.32	62	82	189	0.57	0.60	37	59	192	0.69
4	1.29	16	36	143	0.75	1.20	19	41	174	0.77
