# Supplementary material for: Quantifying selection bias due to unobserved patients in pharmacoepidemiologic studies of severe COVID-19 cohorts
Source: BMC Med Res Methodol. 2026 Jan 16;26:34. doi: 10.1186/s12874-025-02732-w (PMC12896000; doi:10.1186/s12874-025-02732-w)
Supplement: Supplementary file 2 — Supplementary Material 2. [file 12874_2025_2732_MOESM2_ESM.pdf]

Supplementary material: Quantifying selection bias due to unobserved patients in pharmacoepidemiologic studies of severe COVID-19 cohorts.

Marleen Bokern[a*], Christopher T. Rentsch[a], Jennifer Quint[b], Anna Schultze[a], Ian Douglas[a]

*Corresponding author; email: marleen.bokern@lshtm.ac.uk

[a] London School of Hygiene and Tropical Medicine, Keppel Street, London WC1E 7HT, UK

[b] Faculty of Medicine, National Heart & Lung Institute, Imperial College London, London, UK

Supplementary Figure 1 Study diagram 2

Supplementary Figure 2 Directed acyclic graph (DAG) depicting the assumed structure of selection bias 3

Supplementary Figure 3 Unweighted propensity score distribution 3

Supplementary Figure 3 Propensity score distribution after inverse probability of treatment weighting 4

Supplementary Figure 3 Absolute standardised mean differences (SMDs) before and after inverse probability of treatment weighting 5

Supplementary Method 1 Treatment episode estimation 6

Supplementary Method 2 Example calculation, scenario 1 6

Supplementary Table 1. Table of protocol decisions. 7

Supplementary Table 2 2x2 table of hospitalisation and death for ICS group 8

Supplementary Table 3 2x2 table of hospitalisation and death for LABA/LAMA group 8

Supplementary Table 4 Diagnostic checks of scenarios 1-4 9

Supplementary Figure 1 Study diagram

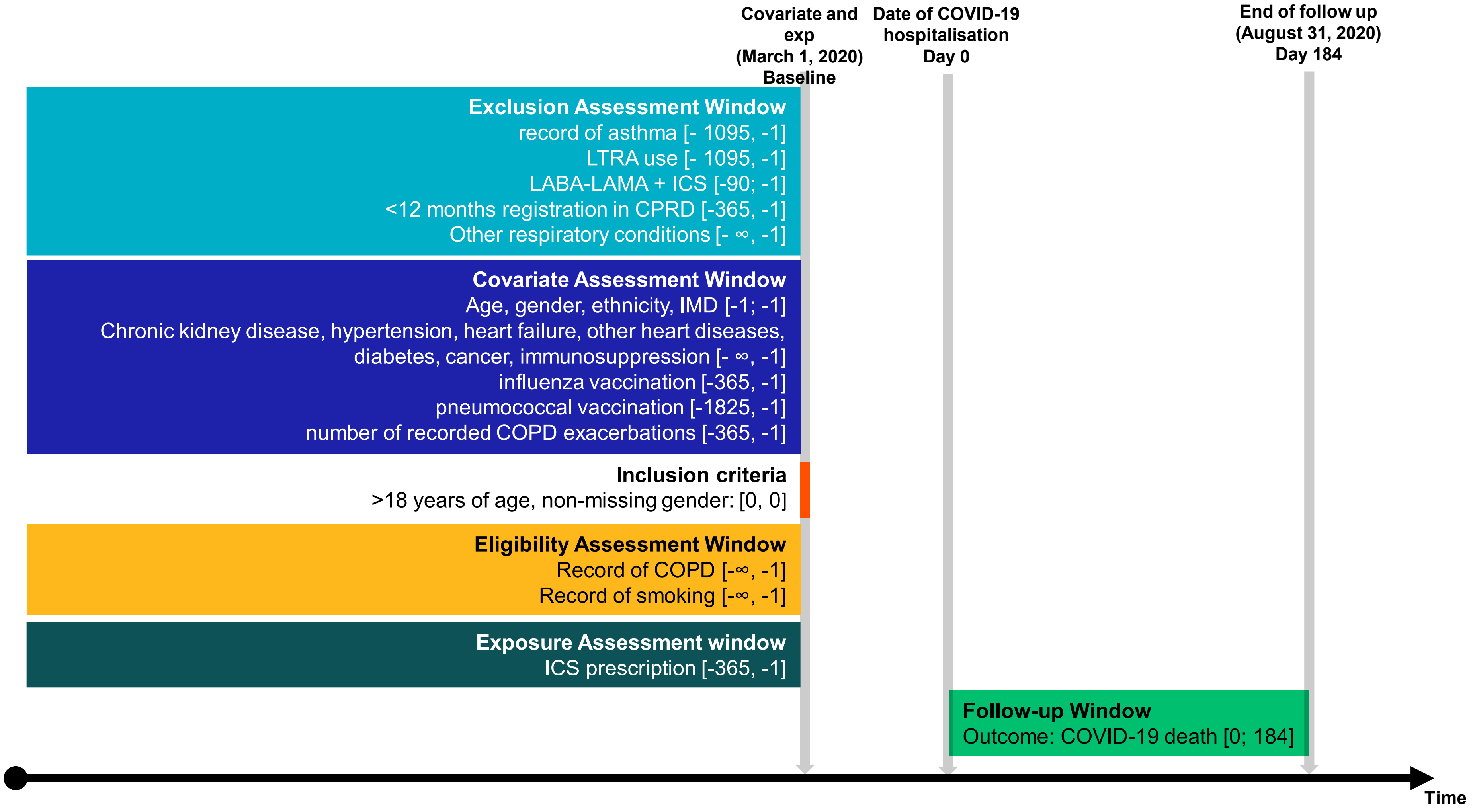

Supplementary Figure 2 Directed acyclic graph (DAG) depicting the assumed structure of selection bias

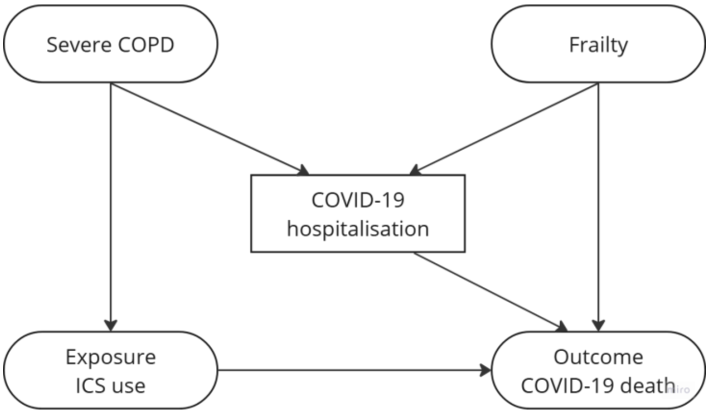

Supplementary Figure 3 Unweighted propensity score distribution

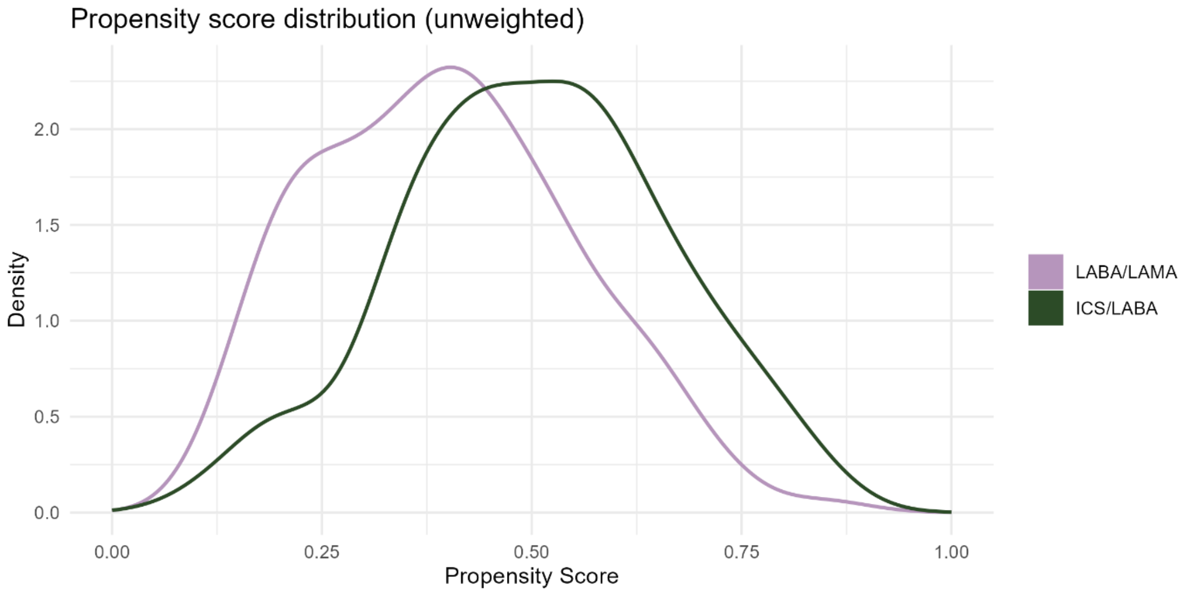

Supplementary Figure 3 Propensity score distribution after inverse probability of treatment weighting

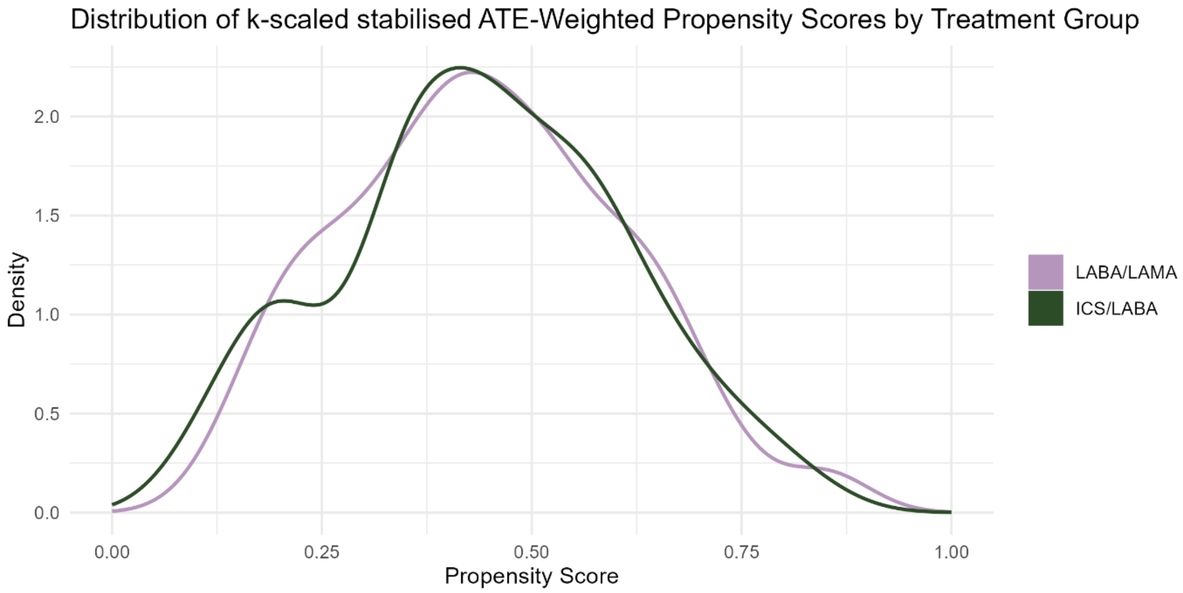

Supplementary Figure 3 Absolute standardised mean differences (SMDs) before and after inverse probability of treatment weighting

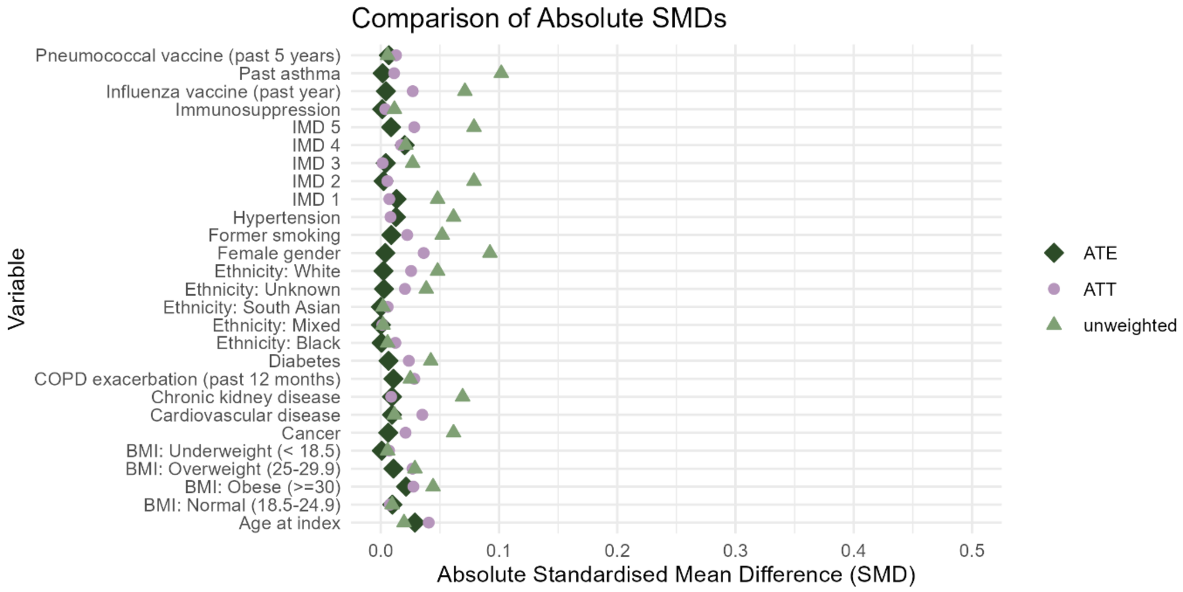

Supplementary Method 1 Treatment episode estimation

Information contained in the quantity variable in the drug issue file in CPRD Aurum was treated as the number of doses per prescription. The estimated number of days' drug supply was calculated by dividing the quantity by the number of daily doses. Where the GP-entered duration was plausible (>7 days and <100 days), that value was used as the exposure duration. Where this was considered implausible, the calculated quantity daily dose was used. Finally, if both were unavailable, the median entered prescription duration for that drug was used. Quantities <10 and >1000 were considered implausible and therefore as missing. If a new prescription was issued before the end of the estimated exposure period of the preceding prescription, the overlapping days were "snowballed" and added on to the end of the calculated exposure period. However, the allowable overlap was capped at 90 days. When a prescription was issued within a 60-day grace period (two times the median duration) of the calculated exposure end of the preceding prescription of the same drug class, these prescriptions were considered as belonging to the same treatment episode and the patient was considered to be using their medication continuously. Discontinuations were thus defined as no new ICS prescription within 60 days of the calculated exposure end date. The discontinuation date was then the end date of the 60-day grace period.

Supplementary Method 2 Example calculation, scenario 1

The odds of death in the hospitalised for each treatment group is as follows:

$$odds_{D=1|E=1,H=1} = \frac{n_{D=1,E=1,H=0}}{n_{D=0,E=1,H=0}} = \frac{42}{65} = 0.65 \tag{1}$$

$$odds_{D=1|E=0,H=1} = \frac{n_{D=1,E=0,H=0}}{n_{D=0,E=0,H=0}} = \frac{50}{83} = 0.60 \tag{2}$$

We assume the odds of death are the same among the non-hospitalised compared to the hospitalised (scenario 1).

As we have data on the number of COVID-19 deaths outside of hospitals by treatment group, we calculate the number of patients with severe COVID-19 who recovered outside of hospital.

$$n_{D=0,E=1,H=0} = \frac{n_{D=1,E=1,H=0}}{odds_{D=1,E=1,H=1}} = \frac{20}{0.65} = 30.95 \approx 31 \tag{3}$$

$$n_{D=0,E=0,H=0} = \frac{n_{D=1,E=0,H=0}}{odds_{D=1,E=0,H=1}} = \frac{22}{0.60} = 36.52 \approx 37 \tag{4}$$

Adding together the observed hospitalisations, the COVID-19 deaths without hospitalisation and the assumed number of recoveries without hospitalisation, we have 158 patients with severe COVID-19 in the ICS group and 192 in the LABA/LAMA group.

Having calculated the number of people with severe COVID-19 who recovered, we can calculate an odds ratio accounting for the people we did not observe in the hospitalisation data.

$$OR = \frac{n_{D=1,E=1} * n_{D=0,E=0}}{n_{D=1,E=0} * n_{D=0,E=1}} = \frac{62 * (83 + 37)}{72 * (65 + 31)} = 1.07 \quad (5)$$

Supplementary Table 1. Table of protocol decisions.

**Protocol Component**	**Description**
Target population	People with COVID-19 severe enough to require hospitalisation, with hospital admission used as a proxy for severe disease.
Eligibility criteria	People diagnosed with COPD before 01st March 2020. Patients were alive and registered in CPRD Aurum at baseline, with ≥12 months' continuous registration prior to baseline. Patients needed to be aged ≥35 and have a record of current or former smoking at any point before baseline.<br>Follow up for the outcome COVID-19 death (recorded using ICD-10 codes U07.1 and U07.2) began on the date of admission for first COVID-19 hospitalisation.
Exposure of Interest and Comparator	Exposure of interest: Use of ICS/LABA at the exposure ascertainment date, defined using estimated treatment start and end dates (Bokern, 2025)<br>Comparator: Use of LABA/LAMA on 01 March 2020, defined using estimated treatment episodes.
Start of follow-up	Date of first COVID-19 hospitalisation
End of follow-up	31st August 2020 or death
Outcome	COVID-19 death, defined as ICD-10 codes U07.1 and U07.2 as a cause of death anywhere on the death certificate in the ONS Death Registry
Analysis plan	Intention-to-treat analogue estimated via comparison of odds of COVID-19 death among individuals in each treatment group. Patients could not switch between treatment groups, and were not censored at discontinuation.<br>Covariates associated with treatment assignment and the outcome were accounted for using inverse probability of treatment weighting, with stabilised ATE weights estimated using propensity scores.

Supplementary Table 2 2x2 table of hospitalisation and death for ICS group

ICS group		Hospitalisation		
		Hospital	No hospital	
**Death**	Death	$n_{D=1,\, H=1}$= 42	$n_{D=1,\, H=0}$ = 20	$n_{D=1}$ = 62
	Survived	$n_{D=0,\, H=1}$ = 65	$n_{D=0,\, H=0}$ = ?	$n_{D=0}$ = ?
		$n_{H=1}$ = 107	$n_{H=0}$ = ?	

Supplementary Table 3 2x2 table of hospitalisation and death for LABA/LAMA group

LABA/LAMA group		Hospitalisation		
		Hospital	No hospital	
**Death**	Death	$n_{D=1,\, H=1}$ = 50	$n_{D=1,\, H=0}$ = 22	$n_{D=1}$ = 72
	Survived	$n_{D=0,\, H=1}$ = 83	$n_{D=0,\, H=0}$ = ?	$n_{D=0}$ = ?
		$n_{H=1}$ = 133	$n_{H=0}$ = ?	

Supplementary Table 4 Diagnostic checks of scenarios 1-4. For totals, decimals were rounded up to the nearest integer.

	ICS					LABA/LAMA				
Scenario	Odds of death among non-hospitalised	n (severe COVID-19, not hospitalised, recovered)	n (severe COVID-19, not hospitalised)	n (severe COVID-19)	p (hospitalisation)	Odds of death among non-hospitalised	n (severe COVID-19, not hospitalised, recovered)	n (severe COVID-19, not hospitalised)	n (severe COVID-19)	p (hospitalisation)
1	0.65	31	51	158	0.68	0.60	37	59	192	0.69
2	1.29	16	36	143	0.75	0.60	37	59	192	0.69
3	0.32	62	82	189	0.57	0.60	37	59	192	0.69
4	1.29	16	36	143	0.75	1.20	19	41	174	0.77
